# Association of first-month beta-hydroxybutyrate levels with subsequent ketogenic diet response in children with drug-resistant epilepsy: a prospective cohort study

**DOI:** 10.3389/fnut.2026.1923059

**Published:** 2026-09-28

**Authors:** Quanzhen Tan, Shuqian Zhang, Yulan Li, Junhong Zhang, Xuting Chang, Yuan Wu, Jie Zhang

**Affiliations:** 1 Children's Medical Center, Department of Pediatric Neurology, Peking University First Hospital, Beijing, China; 2 The Second Qilu Hospital of Shandong University, Jinan, Shandong, China; 3 Peking University First Hospital Ningxia Women and Children's Hospital, Ningxia Hui Autonomous Region Maternal and Child Health Hospital, Yinchuan, Ningxia, China

**Keywords:** beta-hydroxybutyrate, children, clinical nutrition, drug-resistant epilepsy, ketogenic diet therapy, prospective cohort, seizure outcomes

## Abstract

**Background:**

Ketogenic diet therapy (KDT) is an established treatment for children with drug-resistant epilepsy (DRE), but the prognostic value of early blood beta-hydroxybutyrate (BHB) during diet stabilization remains uncertain.

**Methods:**

This single-center prospective cohort study enrolled children with DRE who initiated classical KDT at Peking University First Hospital between June 2023 and January 2025. First-month BHB was defined as the mean of two fasting pre-breakfast BHB measurements obtained during the final week of the first month after KDT initiation. Continuous first-month BHB was analyzed in relation to 3-month seizure reduction. An exploratory group-based analysis used a 3.0 mmol/L cutoff. The primary outcome was 3-month treatment response, defined as ≥50% seizure reduction from baseline. Later response and retention were summarized descriptively.

**Results:**

Of 40 screened children, 38 were analyzed (19 per group). Seizure type differed between groups (*p* = 0.028). At 3 months, response was 42.1% (8/19) in the higher-BHB group and 52.6% (10/19) in the lower-BHB group, with an absolute risk difference of −10.5 percentage points (95% CI, −38.1 to 19.6), risk ratio of 0.80 (95% CI, 0.41–1.58), and unadjusted OR of 0.65 (95% CI, 0.18–2.36). The adjusted OR was 0.64 (95% CI, 0.13–3.09; *p* = 0.575). Continuous BHB showed no clear monotonic association with 3-month seizure reduction (Spearman *ρ* = −0.105; *p* = 0.532). Overall treatment response was 47.4, 39.5, 26.3, and 26.3% at 3, 6, 9, and 12 months, respectively. Adverse events occurred in 13 patients (34.2%) and did not lead to KDT discontinuation.

**Conclusion:**

Continuous first-month BHB showed no clear monotonic association with 3-month seizure reduction; an exploratory 3.0 mmol/L cutoff analysis similarly did not distinguish subsequent responders from non-responders. These findings describe the prognostic value of first-month BHB measured after individualized KDT titration and should not be interpreted as evidence regarding the benefit of deliberately targeting higher BHB levels.

## 1 Introduction

Ketogenic diet therapy (KDT) is a medically supervised nutritional intervention and an important nonpharmacological treatment for children with drug-resistant epilepsy (DRE). Recurrent and uncontrolled seizures can impair cognitive, physical, and psychosocial development, and early seizure control is closely linked to long-term outcomes (1–5). By inducing nutritional ketosis through a high-fat, low-carbohydrate, and adequate-protein dietary pattern, KDT provides alternative metabolic substrates and modulates neuronal excitability through multiple metabolic and signaling pathways. Although KDT has demonstrated clinically meaningful anti-seizure effects in pediatric DRE, with an overall response rate of approximately 36–48.31% (6–8), its implementation requires careful balancing of efficacy, tolerability, and metabolic safety.

Blood beta-hydroxybutyrate (BHB) is the predominant circulating ketone body and is widely used in clinical practice as a practical biomarker of nutritional ketosis during KDT. In many centers, BHB is used not only to confirm adherence to KDT but also to guide dietary titration. Traditionally, higher BHB levels have been interpreted as reflecting a more effective ketogenic state and have therefore been considered desirable for seizure control (9, 10). However, achieving higher peripheral ketone levels often requires increasing the ketogenic ratio, which may increase dietary burden and the risk of gastrointestinal intolerance, hypoglycemia, metabolic acidosis, dyslipidemia, poor growth, and reduced adherence.

Evidence regarding the relationship between BHB levels and KDT efficacy remains inconsistent. Earlier studies suggested that blood BHB may correlate with seizure reduction, whereas more recent studies reported weak, inconsistent, or absent associations (11–15). These discrepancies may be partly explained by methodological heterogeneity, including differences in study design, sample size, dietary protocols, fasting versus non-fasting initiation, and timing of ketone measurement (8, 11–15). In particular, ketone measurements obtained during or near diet initiation may be influenced by acute dietary adaptation, fasting status, and short-term metabolic fluctuations rather than sustained metabolic adaptation (11, 14). In a large pediatric classical KD cohort, Qiao et al. (11) showed that BHB levels rose rapidly after predominantly fasting-based initiation and then stabilized from the end of the first week through 3 months. However, because KDT in the present study was initiated without fasting, metabolic stabilization may require a longer adaptation period. Therefore, the mean BHB level at 1 month was selected as an early stable-phase prognostic exposure to reduce the influence of initiation-related fluctuations and to evaluate whether first-month BHB predicts subsequent KDT response.

The key clinical question is how first-month BHB, measured after early individualized KDT titration, relates to subsequent treatment response in routine practice. This prospective cohort study evaluated whether first-month stable-phase BHB was associated with subsequent KDT response in children with DRE receiving classical KDT. BHB was assessed both as an exploratory 3.0 mmol/L group-based exposure and as a continuous variable.

## 2 Materials and methods

### 2.1 Study design

This was a single-center prospective cohort study conducted at Peking University First Hospital. Children with DRE who initiated classical KDT between June 2023 and January 2025 were prospectively enrolled and followed according to a predefined clinical protocol. The study was registered in the Chinese Clinical Trial Registry (ChiCTR2400079789) and was reported in accordance with the STROBE recommendations for observational studies. We used a 1-month landmark prognostic framework, in which BHB measured at 1 month after KDT initiation was examined in relation to subsequent KDT response.

### 2.2 Participants

Eligible participants were children aged 0–18 years who met the 2010 International League against Epilepsy definition of DRE, defined as failure of two appropriately selected and tolerated antiseizure medications, either as monotherapy or in combination, and who received classical KDT at Peking University First Hospital during the study period.

Patients were excluded if KDT could not be safely or appropriately initiated because of absolute or relative contraindications, including fatty acid oxidation disorders or other relevant metabolic diseases, severe malnutrition, persistent vomiting or diarrhea, severe cardiopulmonary disease, hepatic or renal dysfunction, severe hyperlipidemia, urolithiasis, or severe gastroesophageal reflux. Patients were also excluded if they had a clearly identifiable epileptogenic focus suitable for resective epilepsy surgery, received concomitant treatments that could confound KDT efficacy assessment, such as hormone therapy or vagus nerve stimulation during the early evaluation period, were unable to complete standardized follow-up, or declined participation.

### 2.3 Baseline assessment and follow-up

Before KDT initiation, all participants underwent comprehensive pretreatment screening, including blood and urine metabolic screening, brain magnetic resonance imaging, complete blood count, liver and renal function tests, lipid profile, glucose, electrolytes, echocardiography, and urinary ultrasonography to exclude contraindications and establish baseline safety status.

Baseline clinical variables included sex, age at seizure onset, seizure type, seizure frequency during the 4-week period before KDT initiation, epilepsy syndrome, etiology, developmental status, number of antiseizure medications tried before KDT, and number of antiseizure medications at KDT initiation. KDT-related variables included age at KDT initiation, disease duration at KDT initiation, seizure frequency during follow-up, ketogenic ratio, blood ketone levels, and adverse events.

Participants were followed by telephone and face-to-face visits. During the first week after KDT initiation, patients were monitored daily either by telephone for outpatients or by face-to-face assessment for inpatients. Follow-up was then conducted weekly during weeks 2–4 and monthly thereafter by telephone, with scheduled face-to-face assessments at 3, 6, and 12 months. At each follow-up, seizure type, seizure frequency, KDT adherence, dietary tolerance, and KDT-related adverse events were recorded. Laboratory assessments were performed at 1, 2, 3, 6, and 12 months, including complete blood count, blood glucose, liver and renal function tests, serum electrolytes, lipid profile. Additional examinations, including echocardiography, urinary ultrasonography, and electroencephalography, were conducted at 3, 6, and 12 months when clinically appropriate (Figure 1).

**Figure 1:**
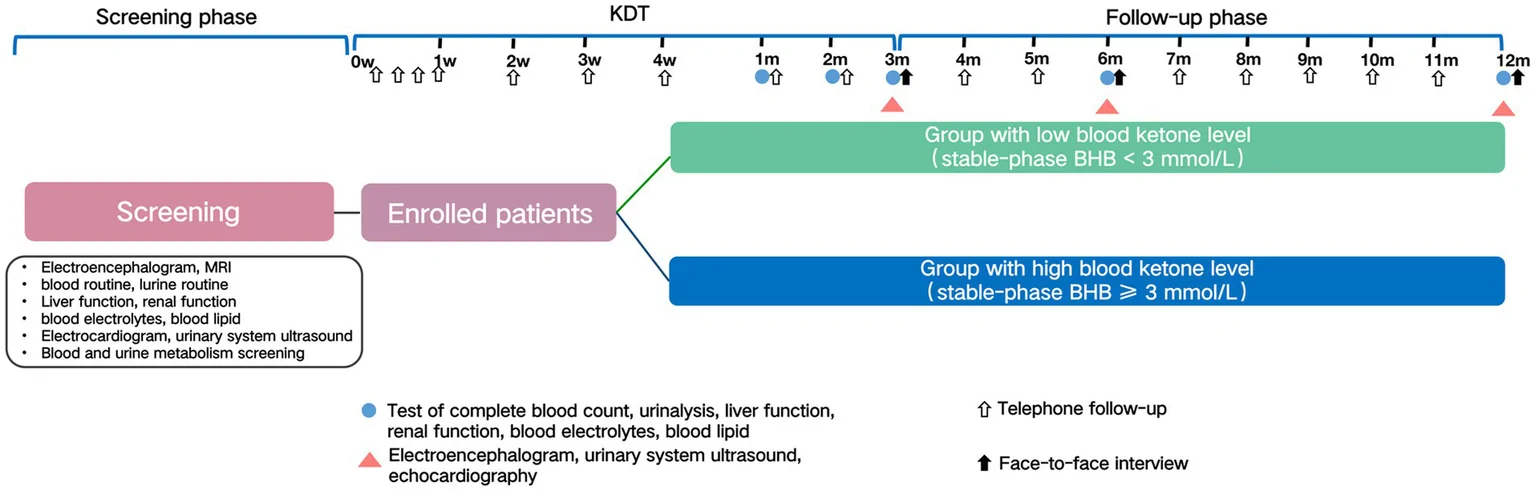
Study design and follow-up schedule. Participants were screened before initiation of KDT, underwent first-month BHB assessment at 1 month, and were followed for treatment response at 3, 6, 9, and 12 months. Scheduled face-to-face assessments were performed at 3, 6, and 12 months, while the 9-month outcome assessment was conducted during routine follow-up.

Seizure frequency was assessed using caregiver-maintained daily seizure diaries. Baseline seizure frequency was calculated from the 4-week period before KDT initiation, and follow-up seizure frequency was calculated from the 4-week period before each scheduled visit. Diary-recorded seizure counts were reviewed during telephone or face-to-face follow-up. For patients with multiple habitual seizure types, the total frequency of recorded habitual seizure events was used consistently for baseline and follow-up comparison. Seizure clusters and epileptic spasms were counted according to clinically recognizable diary-recorded events or clusters rather than by counting each individual spasm when individual events were not reliably separable. Changes in seizure semiology or classification during follow-up were reviewed by the treating neurologist.

### 2.4 Ketogenic diet intervention and clinical management

All participants received standardized classical KDT under medical supervision. KDT was initiated without fasting. The initial ketogenic ratio ranged from 1:1 to 2:1. Thereafter, the ratio was gradually titrated according to a standardized clinical strategy that incorporated seizure control, dietary tolerance, growth status, laboratory safety markers, family adherence, and blood BHB levels. If seizure control improved, the ketogenic ratio was maintained without further escalation. If seizures persisted and the patient tolerated the diet well, with acceptable growth, laboratory safety markers, adherence, and no BHB-related safety concern, the ketogenic ratio was increased stepwise toward higher ratios, up to a maximum of 5:1 when clinically necessary. If adverse effects, poor tolerance, growth concerns, or laboratory abnormalities occurred, the ratio was maintained or reduced as clinically appropriate. Therefore, BHB levels were used as part of metabolic and safety monitoring rather than as the sole trigger for ratio escalation. To minimize confounding of the primary outcome, antiseizure medications were not escalated during the first 3 months of KDT unless clinically necessary for safety. Although clinical indications for KDT differed across epilepsy syndromes, no syndrome-specific BHB target was assigned. Ketogenic ratio adjustment followed the same clinical framework across patients and incorporated seizure control, dietary tolerance, growth status, laboratory safety markers, family adherence, and BHB levels.

### 2.5 Blood BHB measurement and exposure definition

Blood BHB levels were measured using capillary blood testing and recorded in standardized KDT monitoring logbooks. Caregivers were instructed to measure and record BHB according to the clinical monitoring schedule. During the initiation phase, defined as the first week after KDT initiation, BHB was measured two to three times daily and then less frequently as the diet stabilized. When measured twice daily, BHB was assessed before breakfast after overnight fasting and 2 h after dinner; when measured three times daily, BHB was assessed before breakfast after overnight fasting and 2 h after lunch and dinner. At 1 month after KDT initiation, when the ketogenic ratio had reached a relatively stable phase, BHB was measured twice weekly. For this study, “first-month stable-phase BHB” was operationally defined as the mean of two fasting pre-breakfast BHB measurements obtained on two separate days during the final week of the first month after KDT initiation. When more than two fasting pre-breakfast values were recorded during this window, the two measurements with the longest time interval were used to calculate the mean. This one-week mean BHB level was used as the primary exposure to evaluate whether early post-titration BHB was associated with subsequent KDT response.

For exploratory group-based presentation, patients were categorized into a lower-BHB group (<3.0 mmol/L) and a higher-BHB group (≥3.0 mmol/L). This cutoff was selected pragmatically because it lies within the nutritional ketosis range commonly monitored in pediatric KDT practice and is close to stabilized BHB levels reported in previous pediatric classical KDT cohorts (11). The cutoff was used for group-based clinical interpretation, while continuous first-month stable-phase BHB was also analyzed to retain information from the full exposure distribution.

### 2.6 Outcomes

The primary outcome was treatment response at 3 months after KDT initiation, defined as a ≥50% reduction in seizure frequency compared with baseline. Patients with <50% seizure reduction, no change, or increased seizure frequency were classified as non-responders.

Secondary outcomes included response rates at 6, 9, and 12 months; longitudinal response trajectories across follow-up time points; the association between stable-phase BHB as a continuous variable and percentage seizure reduction at 3 months; KDT discontinuation; and KDT-related adverse events.

For response-rate calculations at each follow-up time point, patients who discontinued KDT before that visit were retained in the original denominator and were considered as not achieving treatment response at that visit. This approach was used to reflect the pragmatic outcome of achieving seizure response while remaining on KDT. Because no patient discontinued KDT before the 3-month visit, the primary endpoint was not affected by this rule. For longer-term secondary outcomes at 6, 9, and 12 months, response rates were also summarized among patients who were still receiving KDT at each follow-up visit as a descriptive on-treatment sensitivity analysis. These later analyses were exploratory.

Reasons for KDT discontinuation were classified as ineffectiveness, unsatisfactory effects, or inability to maintain KDT. Ineffectiveness was defined as discontinuation because seizure reduction remained <50%. Unsatisfactory effects were defined as discontinuation despite ≥50% seizure reduction because the improvement did not meet the family’s expected treatment goals or was considered insufficient to justify continuing KDT. Inability to maintain KDT referred to discontinuation because of adherence difficulty, acceptability, dietary burden, or other practical maintenance issues.

KDT-related adverse events included gastrointestinal symptoms, moderate or severe metabolic acidosis (HCO₃^−^ ≤ 13 mmol/L), hypoglycemia (glucose ≤2.8 mmol/L), alanine aminotransferase ≥100 IU/L, hyperlipidemia (triglycerides ≥1.7 mmol/L or total cholesterol ≥5.2 mmol/L), hypokalemia (K < 3.5 mmol/L), hyponatremia (Na < 135 mmol/L), abnormal calcium/phosphorus/magnesium levels, weight loss, and urolithiasis. One patient could contribute more than one type of adverse event, whereas the overall adverse-event incidence was calculated at the patient level as the proportion of patients with at least one adverse event. Event severity was described clinically according to whether the event was transient, required dietary or supportive management, or led to KDT discontinuation.

### 2.7 Statistical analysis

Baseline and clinical characteristics were summarized for the overall cohort and compared between the two stable-phase BHB groups. Continuous variables were presented as median (Q1–Q3), range when non-normally distributed. Categorical variables were presented as frequencies and percentages. Between-group comparisons used the chi-square test, Fisher’s exact test or Fisher–Freeman–Halton extension test for categorical variables and the Mann–Whitney U test for continuous variables, as appropriate.

Sample-size estimation was performed before enrollment based on the limited available evidence regarding the association between blood BHB levels and seizure control in children receiving KDT. In a previous pediatric study, children with relatively higher blood BHB levels during follow-up showed a substantially higher proportion of >50% seizure reduction than those with lower BHB levels (83% vs. 33% using the threshold reported in that study) (13). Using these response proportions for comparison of two independent proportions, with a two-sided α of 0.05 and β of 0.10, the required sample size was estimated to be 12 patients per group using PASS software. Considering an anticipated dropout rate of approximately 20% and the expected KDT case volume at our center, we planned to enroll 20 patients per group, for a total of 40 patients.

The 3.0 mmol/L analysis was used as a group-based comparison for clinical interpretability, and continuous first-month BHB was analyzed to evaluate the exposure without dichotomization. For the primary 3-month binary endpoint, unadjusted effect estimates were reported, including the absolute risk difference, risk ratio, and odds ratio with 95% confidence intervals. Cohen’s h was calculated to quantify the effect size for the difference in response rates.

Multivariable logistic regression was used as an adjusted analysis. Because seizure type and epilepsy syndrome were multicategory variables, they were entered using dummy variables. Therefore, the adjusted model included five predictor parameters in addition to the intercept: one parameter for BHB group, two parameters for seizure type, and two parameters for epilepsy syndrome. Firth penalized logistic regression was additionally performed as a small-sample sensitivity analysis. To assess the influence of major epilepsy syndromes, sensitivity analyses were performed after excluding patients with glucose transporter type 1 deficiency syndrome or infantile epileptic spasm syndrome. Response rates were also summarized descriptively within major syndrome groups.

Spearman correlation analysis was used to examine the association between continuous first-month stable-phase BHB levels and percentage seizure reduction at 3 months. Scatter plots were used to visualize the distribution of first-month stable-phase BHB levels and percentage seizure reduction at 3 months. Slight jittering was applied only to reduce overplotting.

Early seizure response at the 1-month landmark was summarized descriptively according to first-month BHB group. Longitudinal response rates, KDT discontinuation, and treatment retention at 6, 9, and 12 months were summarized descriptively as secondary exploratory outcomes. Response rates were calculated both with discontinued patients retained in the original denominator and among patients who were still receiving KDT at each follow-up visit. Longitudinal response trajectories were displayed using a Sankey diagram showing transitions among response, non-response, and discontinuation states.

All statistical analyses were performed using SPSS version 23.0 and R version 4.2.3. All tests were two-sided, and *p* ≤ 0.05 was considered statistically significant.

### 2.8 Ethics approval and consent to participate

This study was approved by the Biomedical Research Ethics Committee of Peking University First Hospital (approval number: 2023-518). Written informed consent was obtained from the guardians of all participants before enrollment.

## 3 Results

### 3.1 Study population and baseline characteristics

A total of 40 children with DRE were screened for eligibility. Two patients were excluded because of concomitant hormone therapy or vagus nerve stimulation, and 38 patients were included in the final analysis (Figure 2). The median age at seizure onset was 10.5 months (range: 0.5–86.0 months). The median age at KDT initiation was 34.5 months (range: 6–147 months), and the median disease duration at KDT initiation was 21.0 months (range: 2–128 months).

**Figure 2:**
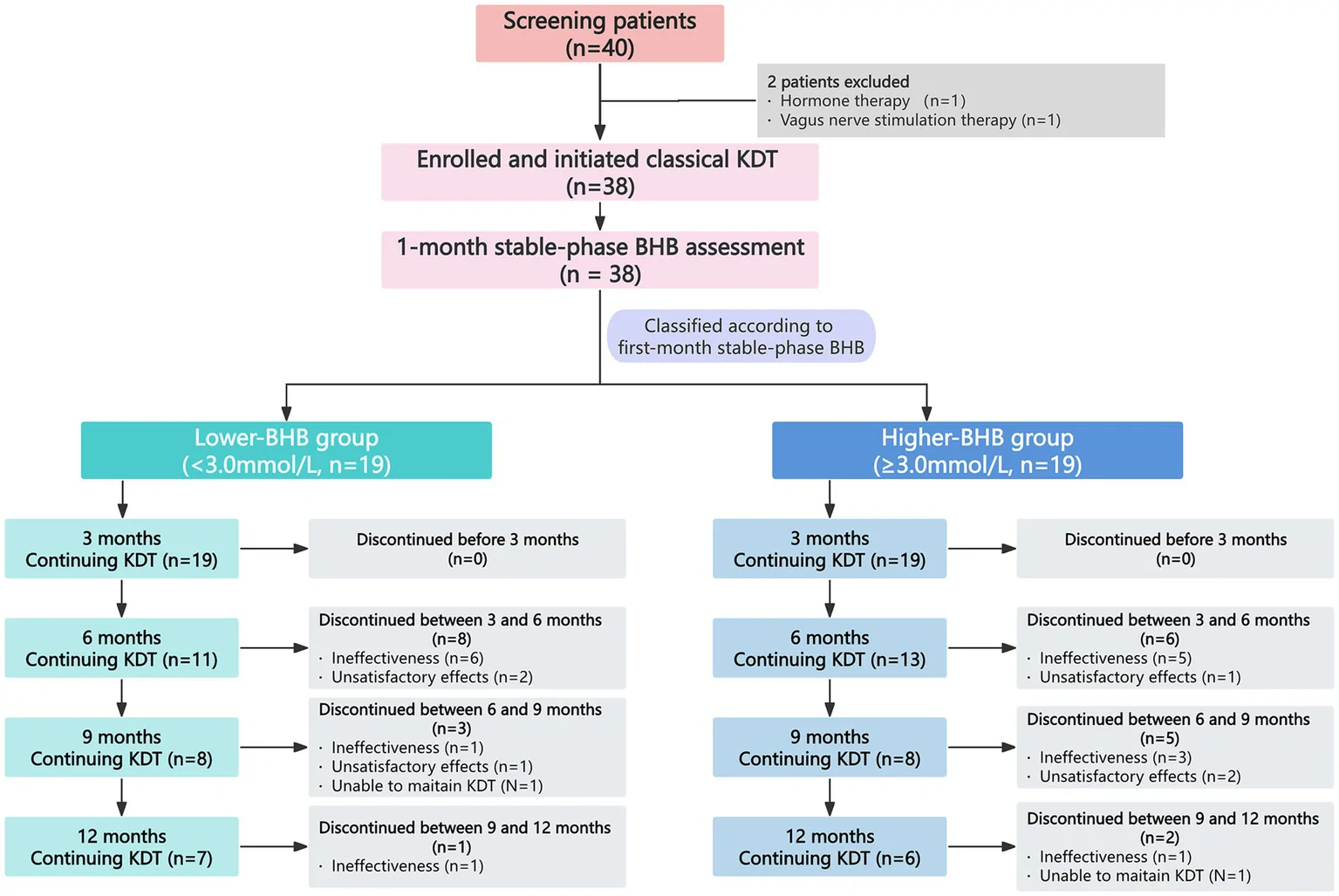
Patient flow diagram. A total of 40 children with drug-resistant epilepsy were screened for eligibility. Two patients were excluded because of concomitant hormone therapy or vagus nerve stimulation, and 38 patients initiated classical KDT. First-month stable-phase BHB was assessed at 1 month after KDT initiation, after which patients were classified into the lower-BHB group (BHB < 3.0 mmol/L, *n* = 19) and higher-BHB group (BHB ≥ 3.0 mmol/L, *n* = 19).

Regarding etiology, 19 patients (50.0%) were classified as having genetic etiology, 2 (5.3%) structural etiology, and 17 (44.7%) other or unknown etiology. Infantile epileptic spasm syndrome was diagnosed in 10 patients (26.3%), and glucose transporter type 1 deficiency syndrome was diagnosed in 7 patients (18.4%). At baseline, 17 patients (44.7%) experienced seizures 10–50 times per day. Seizure types included focal seizures only in 23 patients (60.5%), epileptic spasms only in 8 (21.1%), and other seizure types in 7 (18.4%). Developmental delay was present in 30 patients (78.9%). The median number of anti-seizure medications was 2.5 (range: 2–7) before KDT and 2.0 (range: 0–6) at KDT initiation (Table 1).

**Table 1:** Comparison of baseline characteristics between lower- and higher-BHB groups among analyzed participants (*n* = 38).

Factors	Total (*n* = 38)	Lower-BHB group (*n* = 19)	Higher-BHB group(*n* = 19)	*p* value
Gender				0.515^c^
Male^a^	17 (44.7%)	7 (36.8%)	10 (52.6%)	
Female^a^	21 (55.3%)	12 (63.2%)	9 (47.4%)	
Etiology				1.000^e^
Genetic^a^	19 (50.0%)	10 (52.6%)	9 (47.4%)	
Structural^a^	2 (5.3%)	1 (5.3%)	1 (5.3%)	
Other or unknown^a^	17 (44.7%)	8 (42.1%)	9 (47.4%)	
Epilepsy syndromes been diagnosed				0.063^e^
Infantile epileptic spasm syndrome^a^	10 (26.3%)	2 (10.5%)	8 (42.1%)	
Glucose transporter 1 deficiency syndrome^a^	7 (18.4%)	5 (26.3%)	2 (10.5%)	
Other or undiagnosed syndromes^a^	21 (55.3%)	12 (63.2%)	9 (47.4%)	
Seizure frequency (per day)				0.879^e^
Less than once^a^	6 (15.8%)	4 (21.1%)	2 (10.5%)	
1–10 times^a^	8 (21.1%)	4 (21.1%)	4 (21.1%)	
10–50 times^a^	17 (44.7%)	8 (42.1%)	9 (47.4%)	
More than 50 times^a^	7 (18.4%)	3 (15.8%)	4 (21.1%)	
Type of seizure				**0.028** ^e^
Spasm only^a^	8 (21.1%)	1 (5.3%)	7 (36.8%)	
Focal seizure only^a^	23 (60.5%)	15 (78.9%)	8 (42.1%)	
Others^a^	7 (18.4%)	3 (15.8%)	4 (21.1%)	
Development delay				0.693^d^
No^a^	8 (21.1%)	5 (26.3%)	3 (15.8%)	
Yes^a^	30 (78.9%)	14 (73.7%)	16 (84.2%)	
Age at epilepsy onset (months)^b^	10.5 (2.0–28.5), 0.5–86.0	12.0 (3.0–32.0), 0.5–81.0	6.0 (2.0–28.0), 1.0–86.0	0.455^f^
Age at KDT initiation (months)^b^	34.5 (18.5–84.0), 6.0–147.0	35.0 (31.0–87.0), 11.0–147.0	30.0 (12.0–55.0), 6.0–141.0	0.184^f^
Disease duration at KDT initiation (months)^b^	21.0 (10.0–38.0), 2.0–128.0	23.5 (14.0–41.0), 2.0–123.0	14.5 (10.0–37.0), 4.0–128.0	0.189^f^
ASM use				
Number of ASMs tried before KDT^b^	2.5 (2.0–4.0), 2.0–7.0	2.0 (2.0–5.0), 2.0–7.0	3.0 (2.0–4.0), 2.0–4.0	0.789^f^
Number of ASMs at KDT initiation^b^	2.0 (1.75–3.0), 0.0–6.0	2.0 (1.0–5.0), 0.0–6.0	2.0 (2.0–3.0), 0.0–4.0	0.916^f^

Bold values indicate statistical significance at *p* ≤ 0.05. Data are presented as number (%) or Median (Q1–Q3), range.

BHB, beta-hydroxybutyrate; KDT, ketogenic diet therapy; ASM, antiseizure medication; IQR, interquartile range. *χ^2^* test, Fisher’s exact test and Fisher–Freeman–Halton extension test were used for categorical variables, and the Mann–Whitney U test was used for continuous variables.

^a^Number (%).

^b^Median (Q1–Q3), range.

^c^*χ2* test.

^d^Fisher’s exact test.

^e^Fisher-Freeman–Halton extension test.

^f^Mann–Whitney *U*-test.

### 3.2 Stable-phase BHB groups and baseline comparability

Based on stable-phase BHB levels at 1 month after KDT initiation, 19 patients were categorized into the lower-BHB group (BHB < 3.0 mmol/L) and 19 into the higher-BHB group (BHB ≥ 3.0 mmol/L). For all analyzed patients, first-month stable-phase BHB was calculated from two fasting pre-breakfast BHB measurements obtained on two separate days during the final week of the first month after KDT initiation. No missing BHB values were present for this exposure definition. Seizure type differed between groups (*p* = 0.028), with a higher proportion of epileptic spasms in the higher-BHB group and a higher proportion of focal seizures in the lower-BHB group. Other baseline characteristics were broadly comparable between groups, including sex, etiology, epilepsy syndrome, baseline seizure frequency, developmental delay, age at seizure onset, age at KDT initiation, disease duration before KDT, and number of antiseizure medications (Table 1). Because seizure type may influence KDT response, it was included in the adjusted analyses.

### 3.3 Early titration and treatment response according to stable-phase BHB group

All patients initiated KDT using a standardized non-fasting protocol, with an initial prescribed ketogenic ratio ranging from 1:1 to 2:1. During early treatment, the ketogenic ratio was adjusted according to seizure control, dietary tolerance, growth status, laboratory safety findings, adherence, and BHB-related metabolic and safety monitoring. At KDT initiation, the prescribed ketogenic ratios were 1:1 to <2:1 in 34 patients and 2:1 to <3:1 in 4 patients. At the 1-month landmark, the prescribed ketogenic ratios were distributed as follows: 1:1 to <2:1 in 17 patients, 2:1 to <3:1 in 18 patients, and 3:1 to <4:1 in 3 patients. At the 3-month primary evaluation, the corresponding numbers were 12, 18, 6, and 2 patients for 1:1 to <2:1, 2:1 to <3:1, 3:1 to <4:1, and ≥4:1, respectively. From KDT initiation to the 1-month landmark, the prescribed ketogenic ratio increased in 29 patients, remained unchanged in 7 patients, and decreased in 2 patients. From 1 month to 3 months, the prescribed ketogenic ratio increased in 19 patients, remained unchanged in 17 patients, and decreased in 2 patients. These data summarize the individualized dietary titration process before and after first-month BHB assessment.

At the 1-month landmark, early seizure response was summarized descriptively to characterize the clinical status around the time of BHB assessment. Overall, 11 of 38 patients (28.9%) achieved ≥50% seizure reduction from baseline at 1 month. The 1-month response rate was 26.3% (5/19) in the higher-BHB group and 31.6% (6/19) in the lower-BHB group.

No patients discontinued KDT before the 3-month primary evaluation, and all 38 patients contributed to the primary endpoint analysis. At the 3-month primary endpoint, the overall response rate was 47.4% (18/38). The response rate was 42.1% (8/19) in the higher-BHB group and 52.6% (10/19) in the lower-BHB group. Using the lower-BHB group as the reference, the absolute risk difference was −10.5 percentage points (95% CI, −38.1 to 19.6), the risk ratio was 0.80 (95% CI, 0.41–1.58), and the unadjusted OR was 0.65 (95% CI, 0.18–2.36), corresponding to a small effect size (Cohen’s *h* = 0.21; *p* = 0.75). In the adjusted logistic regression model, which included five predictor parameters in addition to the intercept, the adjusted OR for higher first-month BHB was 0.64 (95% CI, 0.13–3.09; *p* = 0.575). Firth penalized logistic regression yielded a similar estimate (OR = 0.69; 95% CI, 0.16–2.88; *p* = 0.609).

After the 3-month primary evaluation, treatment discontinuation increased over time. By 6, 9, and 12 months, 36.8% (14/38), 57.9% (22/38), and 65.8% (25/38) of patients had discontinued KDT, respectively. Insufficient efficacy was the most common reason for discontinuation. Between 3 and 6 months, 14 patients discontinued KDT, including 11 because of insufficient efficacy and 3 because of partial response with clinically unsatisfactory seizure control. Between 6 and 9 months, 8 additional patients discontinued KDT, including 4 because of insufficient efficacy, 3 because of partial response with clinically unsatisfactory seizure control, and 1 because of inability to maintain KDT. Between 9 and 12 months, 3 additional patients discontinued KDT, including 2 because of insufficient efficacy and 1 because of inability to maintain KDT.

Response rates at later follow-up points were summarized descriptively. In the higher- versus lower-BHB groups, response rates were 36.8% versus 42.1% at 6 months, 26.3% versus 26.3% at 9 months, and 26.3% versus 26.3% at 12 months, respectively (Figure 3).

**Figure 3:**
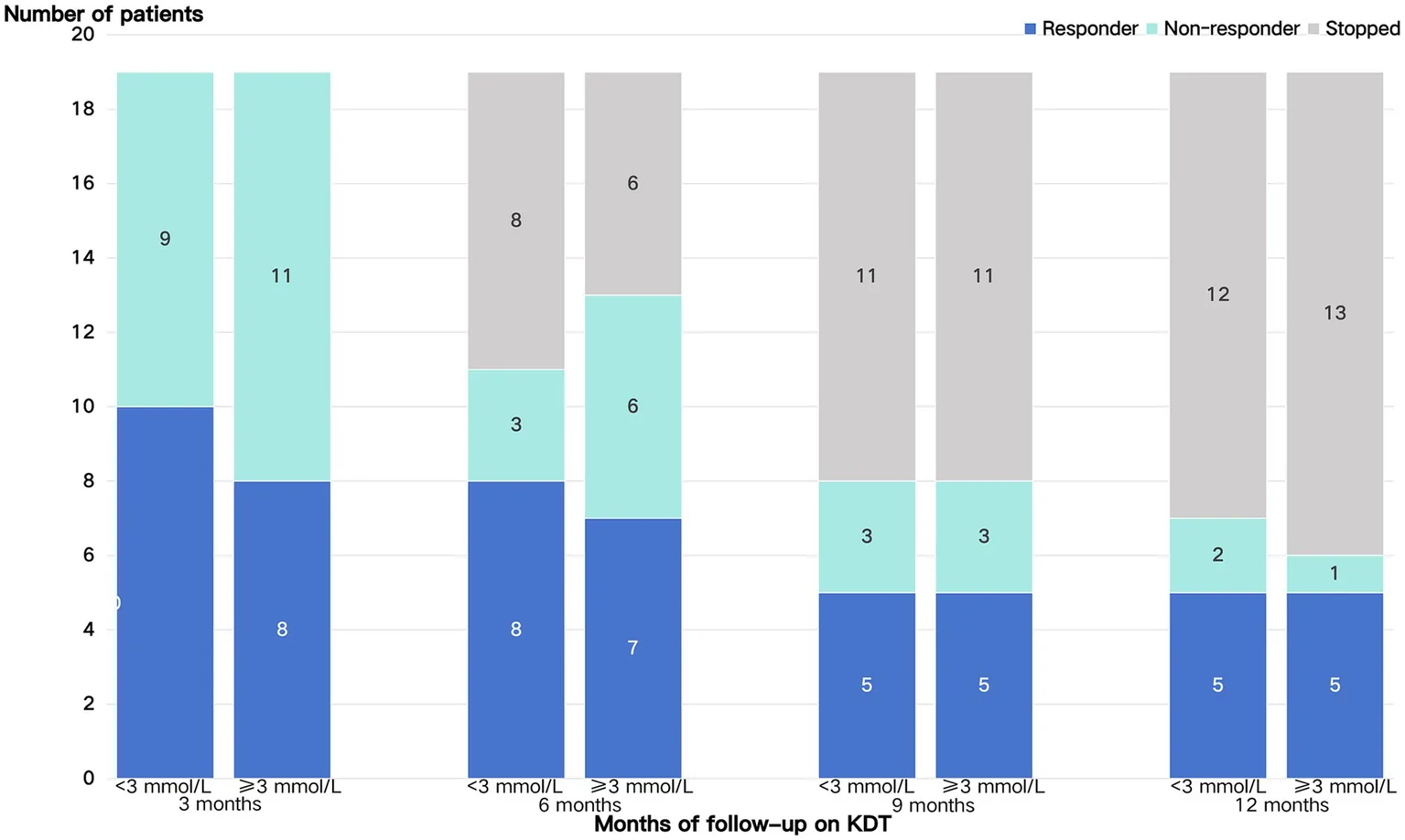
Response status at 3, 6, 9, and 12 months according to stable-phase BHB group. Stacked bar charts show the numbers of responders, non-responders, and patients who discontinued KDT in each first-month stable-phase BHB group at each follow-up time point. Treatment response was defined as a ≥ 50% reduction in seizure frequency from baseline. For response-rate calculations, patients who discontinued KDT were retained in the original denominator and were not counted as responders at subsequent follow-up time points. The stacked bars display discontinued patients as a separate descriptive category.

In sensitivity analyses excluding patients with glucose transporter type 1 deficiency syndrome or infantile epileptic spasm syndrome, response patterns were descriptively similar to the main analysis. After excluding glucose transporter type 1 deficiency syndrome, the 3-month response rate was 41.2% (7/17) in the higher-BHB group and 42.9% (6/14) in the lower-BHB group. After excluding infantile epileptic spasm syndrome, the corresponding rates were 45.5% (5/11) and 58.8% (10/17), respectively.

### 3.4 Continuous-variable analysis

To reduce reliance on dichotomization, first-month stable-phase BHB was also analyzed as a continuous variable. Spearman correlation analysis did not detect a clear monotonic association between first-month stable-phase BHB levels and percentage seizure reduction at 3 months (*ρ* = −0.105, *p* = 0.532; *n* = 38). The scatter plot showed no clear visual pattern between first-month stable-phase BHB and 3-month seizure reduction (Figure 4). Together, the continuous-variable analysis and exploratory group-based comparison did not detect a clear association between first-month stable-phase BHB and 3-month seizure reduction.

**Figure 4:**
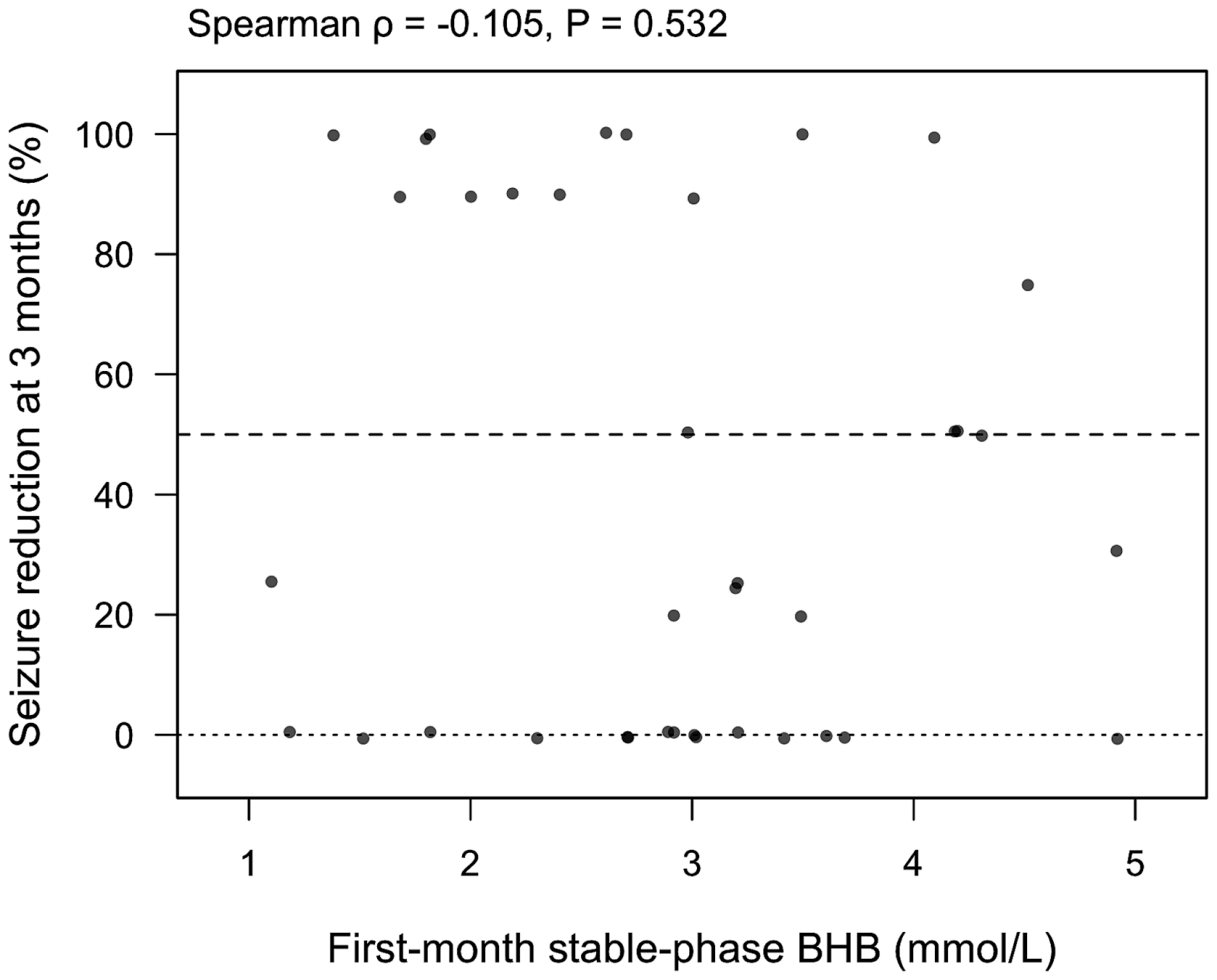
Association between stable-phase BHB levels and seizure reduction at 3 months after KDT initiation. The scatter plot shows the distribution of first-month stable-phase blood BHB levels and percentage reduction in seizure frequency at 3 months. Slight jittering was applied to reduce overplotting. Spearman correlation analysis did not detect a clear monotonic association between first-month stable-phase BHB and 3-month seizure reduction (*ρ* = −0.105, *p* = 0.532, *n* = 38).

### 3.5 Exploratory longitudinal response and treatment retention

Outcomes at 6, 9, and 12 months were analyzed as exploratory secondary outcomes. When patients who discontinued KDT were retained in the original denominator and counted as non-responders, the overall response rate was 39.5% (15/38) at 6 months and 26.3% (10/38) at both 9 and 12 months. Response rates in the higher- versus lower-BHB groups were 36.8% (7/19) versus 42.1% (8/19) at 6 months, 26.3% (5/19) versus 26.3% (5/19) at 9 months, and 26.3% (5/19) versus 26.3% (5/19) at 12 months, respectively.

Treatment retention declined over time, with 63.2% (24/38), 42.1% (16/38), and 34.2% (13/38) of patients remaining on KDT at 6, 9, and 12 months, respectively. As a descriptive on-treatment sensitivity analysis among patients who continued KDT, response rates in the higher- versus lower-BHB groups were 53.8% (7/13) versus 72.7% (8/11) at 6 months, 62.5% (5/8) versus 62.5% (5/8) at 9 months, and 83.3% (5/6) versus 71.4% (5/7) at 12 months, respectively. These on-treatment analyses were descriptive because patients remaining on KDT represented a selected subgroup. The Sankey diagram was used to visualize individual transitions among response, non-response, and discontinuation states across follow-up (Figure 5).

**Figure 5:**
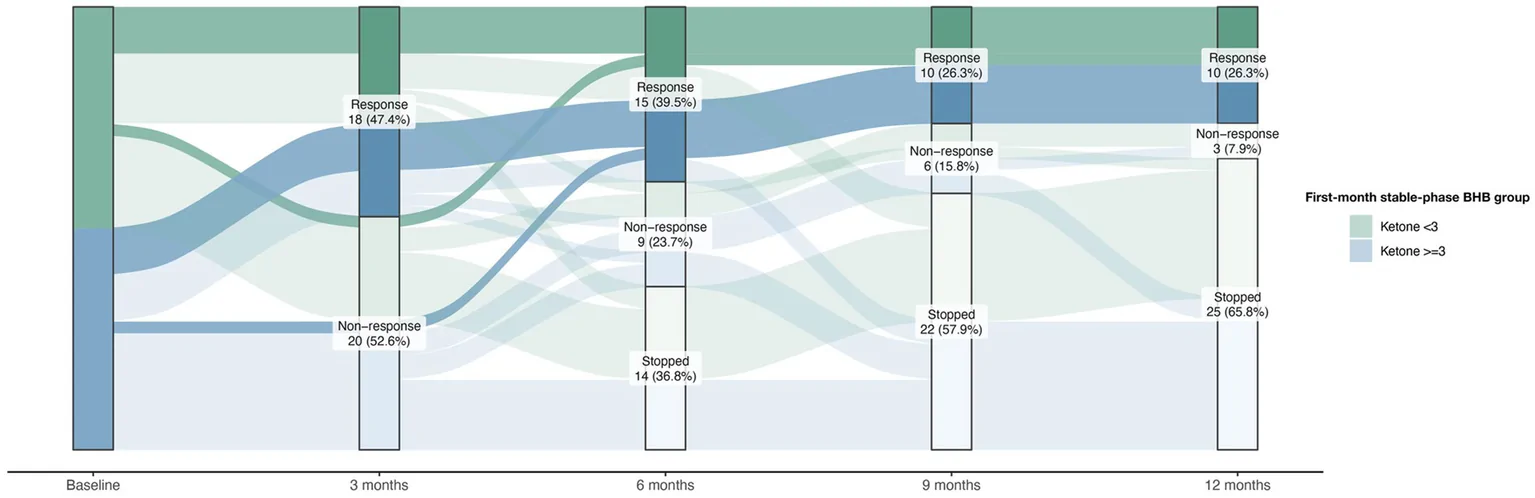
Longitudinal response trajectories during KDT. The Sankey diagram illustrates individual transitions among response, non-response, and discontinuation states from 3 to 12 months after KDT initiation. Link widths are proportional to the number of patients transitioning between states. Link colors indicate first-month stable-phase BHB group. The diagram was used as a descriptive visualization of treatment trajectories.

### 3.6 Adverse events

Adverse-event analyses were descriptive. Overall, 13 patients (34.2%) experienced at least one KDT-related adverse event, and one patient could contribute more than one event type. The most common events were transient hypoglycemia (*n* = 7; all occurred within the first month after KDT initiation), gastrointestinal symptoms (*n* = 6; all occurred within the first month), and hyperlipidemia (*n* = 4; all occurred after the 1-month BHB assessment). All events were transient and were managed with dietary adjustment, clinical monitoring, or supportive care; no adverse event led to KDT discontinuation. The patient-level incidence of adverse events was 21.1% (4/19) in the higher-BHB group and 47.4% (9/19) in the lower-BHB group. This comparison was interpreted descriptively because dietary tolerance, laboratory safety findings, and growth concerns were considered during ketogenic-ratio titration.

## 4 Discussion

In this prospective cohort of children with DRE receiving classical KDT, first-month stable-phase BHB ≥ 3.0 mmol/L did not clearly distinguish subsequent 3-month responders from non-responders after individualized early KDT titration. This pattern was consistent across the group-based comparison, adjusted analysis, Firth sensitivity analysis, and continuous BHB analysis. A key feature of the study is that first-month BHB was measured after early clinical titration, when dietary ratio, seizure response, tolerability, safety findings, and adherence had already shaped management. Thus, the findings describe the prognostic value of an early post-titration BHB measurement in routine KDT care. Longer-term follow-up showed declining treatment retention over time, with most discontinuations related to insufficient efficacy or clinically unsatisfactory seizure control and none attributed to adverse events.

Traditionally, higher ketone levels have been considered indicative of a more effective ketogenic state and, consequently, improved seizure control (2, 12, 13). However, evidence regarding the relationship between BHB levels and seizure control across the course of KDT remains inconsistent. While early studies, such as that by van Delft et al. (12) reported a positive correlation between BHB levels and seizure reduction, later studies have reported weaker or inconsistent associations (14, 15). Qiao et al. (11) in a retrospective cohort of 300 children, identified a physiological BHB range of 1.1–4.9 mmol/L but did not demonstrate a clear dose–response relationship with treatment efficacy. In addition, prior studies from our center have suggested that KDT efficacy may be influenced by factors such as treatment retention, disease characteristics, epilepsy syndrome, and broader clinical heterogeneity rather than ketone levels alone (8, 16). Our findings add prospective data focused on first-month BHB after non-fasting KDT initiation and support the view that peripheral BHB should be interpreted within the broader clinical context of KDT management.

The present study evaluated first-month stable-phase BHB as an early prognostic exposure for subsequent KDT response. Later BHB values may fluctuate with dietary adherence, intercurrent illness, reduced intake, and other clinical events during long-term KDT. Future studies with repeated BHB measurements throughout follow-up may clarify whether dynamic BHB patterns improve response prediction beyond a single early post-titration measurement.

Clinically, our findings suggest that first-month stable-phase BHB should be interpreted as one component of KDT monitoring rather than as an isolated predictor of subsequent seizure response. In this cohort, higher first-month BHB did not clearly distinguish later responders from non-responders, while dietary titration was shaped by seizure response, tolerance, laboratory safety, growth status, and family adherence. This interpretation is consistent with international recommendations emphasizing structured monitoring, multidisciplinary support, and individualized management during pediatric KDT, particularly when resources and follow-up capacity vary across clinical settings (17, 18).

The relationship between first-month stable-phase BHB category and ketogenic ratio advancement should be interpreted in the context of individualized titration. The prescribed ketogenic ratio increased in most patients during the first month, but ratio distributions remained heterogeneous at 1 and 3 months. A lower BHB level may coexist with adequate response at a lower ratio or slower advancement for tolerability, whereas a higher BHB level may reflect metabolic responsiveness, tolerance of further escalation, or continued titration in patients requiring additional seizure control. These findings support interpreting BHB together with the clinical context of dietary titration, rather than as a standalone measure of dietary intensity.

From a mechanistic perspective, several biological considerations may help explain why peripheral BHB is an incomplete proxy for clinical response. The anti-seizure effects of KDT are multifactorial and may involve modulation of neuronal excitability through glutamatergic, GABAergic, ATP-sensitive potassium channel, and adenosine-related pathways (9, 19), as well as changes in mitochondrial function and cerebral energy metabolism (20–22). BHB may also act as a metabolic signaling molecule through histone deacetylase inhibition, inflammatory pathway modulation, mitochondrial effects, and oxidative-stress regulation (23, 24). Peripheral BHB may incompletely reflect cerebral ketone availability or utilization, which depends on monocarboxylate transporter activity, blood–brain barrier function, and tissue-level metabolic adaptation (25–27). KDT may further exert effects through BHB-independent pathways, including gut microbiota-related mechanisms (28). These mechanisms provide biological context for future studies but were not directly assessed in this clinical cohort.

Safety findings were broadly consistent with previous pediatric KDT reports (2, 8, 29). Adverse events occurred in 34.2% (13/38) of patients, mainly including transient hypoglycemia, gastrointestinal symptoms, and hyperlipidemia. These events were generally transient and did not lead to KDT discontinuation. Because safety findings were incorporated into dietary titration, adverse-event comparisons were interpreted descriptively. This study has several strengths. The prospective design reduced recall and selection biases compared with retrospective studies, and the use of a first-month mean BHB improved exposure assessment. The standardized KDT protocol, scheduled follow-up, and restriction of antiseizure medication escalation during the first 3 months strengthened internal validity. The study also evaluated BHB using both a clinically interpretable threshold and continuous-variable analysis.

Several limitations should be acknowledged. First, the sample size limited the precision of adjusted and subgroup analyses. Second, first-month BHB was measured after individualized ketogenic-ratio titration rather than under predefined BHB targets or fixed-ratio protocols. Because early clinical response, tolerability, safety findings, and adherence contributed to dietary titration before the 1-month BHB assessment, confounding by indication and potential reverse causation are inherent to this design. Third, first-month stable-phase BHB was an operational exposure definition based on two fasting pre-breakfast measurements during the final week of the first month. Later time-varying BHB changes were not systematically analyzed. Future multicenter studies with larger samples and repeated longitudinal BHB measurements are needed to evaluate dynamic BHB patterns and their relationship with long-term outcomes.

## 5 Conclusion

In conclusion, in this prospective cohort of children with DRE receiving classical KDT, continuous first-month BHB showed no clear monotonic association with 3-month seizure reduction. An exploratory 3.0 mmol/L cutoff analysis similarly did not distinguish subsequent responders from non-responders. These findings support integrating early BHB with clinical response, dietary tolerance, metabolic safety, growth, and adherence during individualized KDT management.

## Data Availability

The raw data supporting the conclusions of this article will be made available by the authors, without undue reservation.

## References

[1] Freitag H Tuxhorn I. Cognitive function in preschool children after epilepsy surgery: rationale for early intervention. Epilepsia. (2005) 46:561–7. doi: 10.1111/j.0013-9580.2005.03504.x, 15816951

[2] Wells J Swaminathan A Paseka J Hanson C. Efficacy and safety of a ketogenic diet in children and adolescents with refractory epilepsy—a review. Nutrients. (2020) 12:1809. doi: 10.3390/nu12061809, 32560503 PMC7353240

[3] Nordli DR Kuroda MM Carroll J Koenigsberger DY Hirsch LJ Bruner HJ . Experience with the ketogenic diet in infants. Pediatrics. (2001) 108:129–33. doi: 10.1542/peds.108.1.129 11433065

[4] Alharbi HM Pinchefsky EF Tran M-A Salazar Cerda CI Parokaran Varghese J Kamino D . Seizure burden and neurologic outcomes after neonatal encephalopathy. Neurology. (2023) 100:e1976–84. doi: 10.1212/WNL.0000000000207202, 36990719 PMC10186227

[5] Kim H-J Kim M-J Kim EA Kwon O Nam YS Yum M-S . Childhood-onset epilepsy: longitudinal seizure outcomes in a large single-center cohort. Epilepsy Res. (2025) 217:107622. doi: 10.1016/j.eplepsyres.2025.107622, 40651169

[6] Devi N Madaan P Kandoth N Bansal D Sahu JK. Efficacy and safety of dietary therapies for childhood drug-resistant epilepsy: a systematic review and network Meta-analysis. JAMA Pediatr. (2023) 177:258–66. doi: 10.1001/jamapediatrics.2022.5648, 36716045 PMC9887534

[7] Pizzo F Collotta AD Di Nora A Costanza G Ruggieri M Falsaperla R. Ketogenic diet in pediatric seizures: a randomized controlled trial review and meta-analysis. Expert Rev Neurother. (2022) 22:169–77. doi: 10.1080/14737175.2022.2030220, 35144527

[8] Zhang J Gu Q Wu Y Xie H Wei C Zhang Y . Confirmatory study on the retention rate of ketogenic diet therapy and related factors in children with drug-resistant epilepsy: a single-center cohort study. Epilepsia Open. (2025) 10:1147–60. doi: 10.1002/epi4.70077, 40538087 PMC12362158

[9] Simeone TA Simeone KA Stafstrom CE Rho JM. Do ketone bodies mediate the anti-seizure effects of the ketogenic diet? Neuropharmacology. (2018) 133:233–41. doi: 10.1016/j.neuropharm.2018.01.011, 29325899 PMC5858992

[10] Juge N Gray JA Omote H Miyaji T Inoue T Hara C . Metabolic control of vesicular glutamate transport and release. Neuron. (2010) 68:99–112. doi: 10.1016/j.neuron.2010.09.002, 20920794 PMC2978156

[11] Qiao X Ye Z Wen J Lin S Cao D Chen L . Exploring physiological beta-hydroxybutyrate level in children treated with the classical ketogenic diet for drug-resistant epilepsy. Acta Epileptol. (2025) 7:10. doi: 10.1186/s42494-024-00199-8, 40217541 PMC11960278

[12] Van Delft R Lambrechts D Verschuure P Hulsman J Majoie M. Blood beta-hydroxybutyrate correlates better with seizure reduction due to ketogenic diet than do ketones in the urine. Seizure. (2010) 19:36–9. doi: 10.1016/j.seizure.2009.10.009, 19962324

[13] Gilbert DL Pyzik PL Freeman JM. The ketogenic diet: seizure control correlates better with serum β-hydroxybutyrate than with urine ketones. J Child Neurol. (2000) 15:787–90. doi: 10.1177/088307380001501203, 11198492

[14] Buchhalter JR D’Alfonso S Connolly M Fung E Michoulas A Sinasac D . The relationship between D -beta-hydroxybutyrate blood concentrations and seizure control in children treated with the ketogenic diet for medically intractable epilepsy. Epilepsia Open. (2017) 2:317–21. doi: 10.1002/epi4.12058 PMC5862113 29588960

[15] Teng L Lee VW Murugesu S Lee J Ibrahim NS Ishak MF . Glycemic biomarkers in children with drug-resistant epilepsy on various types of ketogenic diet therapies: a cross-sectional study. Epilepsia. (2022) 63:2011–23. doi: 10.1111/epi.17292, 35527506

[16] Zhang J Chen G Wang J Jiang Y Yang Z Xu K . Efficacy of the ketogenic diet on ACTH- or corticosteroid-resistant infantile spasm: a multicentre prospective control study. Epileptic Disord. (2021) 23:337–45. doi: 10.1684/epd.2021.1256, 33772508

[17] Kossoff EH Al-Macki N Cervenka MC Kim HD Liao J Megaw K . What are the minimum requirements for ketogenic diet services in resource-limited regions? Recommendations from the international league against epilepsy task force for dietary therapy. Epilepsia. (2015) 56:1337–42. doi: 10.1111/epi.13039, 26033161

[18] Kossoff EH Zupec-Kania BA Auvin S Ballaban-Gil KR Christina Bergqvist AG Blackford R . Optimal clinical management of children receiving dietary therapies for epilepsy: updated recommendations of the international ketogenic diet study group. Epilepsia Open. (2018) 3:175–92. doi: 10.1002/epi4.12225, 29881797 PMC5983110

[19] Masino S. A. Rho J. M. Mechanisms of Ketogenic Diet Action. Jasper’s Basic Mechanisms of the Epilepsies. Bethesda, MD: National Center for Biotechnology Information (US) (2012). 22787591

[20] Lutas A Yellen G. The ketogenic diet: metabolic influences on brain excitability and epilepsy. Trends Neurosci. (2013) 36:32–40. doi: 10.1016/j.tins.2012.11.005, 23228828 PMC3534786

[21] Gano LB Patel M Rho JM. Ketogenic diets, mitochondria, and neurological diseases. J Lipid Res. (2014) 55:2211–28. doi: 10.1194/jlr.R048975, 24847102 PMC4617125

[22] Newmaster K Zhu Z Bolt E Chang RJ Day C Mhanna A . A review of the multi-systemic complications of a ketogenic diet in children and infants with epilepsy. Children. (2022) 9:1372. doi: 10.3390/children9091372, 36138681 PMC9498174

[23] Shimazu T Hirschey MD Newman J He W Shirakawa K Le Moan N . Suppression of oxidative stress by β-hydroxybutyrate, an endogenous histone deacetylase inhibitor. Science. (2013) 339:211–4. doi: 10.1126/science.1227166, 23223453 PMC3735349

[24] Youm Y-H Nguyen KY Grant RW Goldberg EL Bodogai M Kim D . The ketone metabolite β-hydroxybutyrate blocks NLRP3 inflammasome–mediated inflammatory disease. Nat Med. (2015) 21:263–9. doi: 10.1038/nm.3804, 25686106 PMC4352123

[25] Ramezani M Fernando M Eslick S Asih PR Shadfar S Bandara EMS . Ketone bodies mediate alterations in brain energy metabolism and biomarkers of Alzheimer’s disease. Front Neurosci. (2023) 17:1297984. doi: 10.3389/fnins.2023.1297984, 38033541 PMC10687427

[26] Rubio C López-Landa A Romo-Parra H Rubio-Osornio M. Impact of the ketogenic diet on neurological diseases: a review. Life. (2025) 15:71. doi: 10.3390/life15010071, 39860011 PMC11767209

[27] Chasseigneaux S Cochois-Guégan V Lecorgne L Lochus M Nicolic S Blugeon C . Fasting upregulates the monocarboxylate transporter MCT1 at the rat blood-brain barrier through PPAR δ activation. Fluids Barriers CNS. (2024) 21:33. doi: 10.1186/s12987-024-00526-8, 38589879 PMC11003008

[28] Olson CA Vuong HE Yano JM Liang QY Nusbaum DJ Hsiao EY. The gut microbiota mediates the anti-seizure effects of the ketogenic diet. Cell. (2018) 173:1728–41. doi: 10.1016/j.cell.2018.04.027, 29804833 PMC6003870

[29] Ray S Nathan J Godhia M. Efficacy and tolerability of classical and polyunsaturated fatty acids ketogenic diet in controlling paediatric refractory epilepsy—a randomized study. Epilepsy Res. (2024) 204:107395. doi: 10.1016/j.eplepsyres.2024.107395, 38908324

